# Antifungal prophylaxis with posaconazole vs. fluconazole or itraconazole in pediatric patients with neutropenia

**DOI:** 10.1007/s10096-015-2340-y

**Published:** 2015-02-14

**Authors:** M. Döring, M. Eikemeier, K. M. Cabanillas Stanchi, U. Hartmann, M. Ebinger, C.-P. Schwarze, A. Schulz, R. Handgretinger, I. Müller

**Affiliations:** 1 Department I—General Paediatrics, Hematology/Oncology, University Hospital Tuebingen, Children’s Hospital, Hoppe-Seyler-Str. 1, 72076 Tuebingen, Germany; 2 Department of Pediatric Hematology and Oncology, University Hospital Ulm, Children’s Hospital, Albert-Einstein-Allee 23, 89081 Ulm, Germany; 3 Pharmacy, University Hospital Tuebingen, Children’s Hospital, Hoppe-Seyler-Str. 1, 72076 Tuebingen, Germany; 4 Clinic of Pediatric Hematology and Oncology, University Medical Center Hamburg-Eppendorf, Martinistr. 52, 20246 Hamburg, Germany

## Abstract

Pediatric patients with hemato-oncological malignancies and neutropenia resulting from chemotherapy have a high risk of acquiring invasive fungal infections. Oral antifungal prophylaxis with azoles, such as fluconazole or itraconazole, is preferentially used in pediatric patients after chemotherapy. During this retrospective analysis, posaconazole was administered based on favorable results from studies in adult patients with neutropenia and after allogeneic hematopoietic stem cell transplantation. Retrospectively, safety, feasibility, and initial data on the efficacy of posaconazole were compared to fluconazole and itraconazole in pediatric and adolescent patients during neutropenia. Ninety-three pediatric patients with hemato-oncological malignancies with a median age of 12 years (range 9 months to 17.7 years) that had prolonged neutropenia (>5 days) after chemotherapy or due to their underlying disease, and who received fluconazole, itraconazole, or posaconazole as antifungal prophylaxis, were analyzed in this retrospective single-center survey. The incidence of invasive fungal infections in pediatric patients was low under each of the azoles. One case of proven aspergillosis occurred in each group. In addition, there were a few cases of possible invasive fungal infection under fluconazole (*n* = 1) and itraconazole (*n* = 2). However, no such cases were observed under posaconazole. The rates of potentially clinical drug-related adverse events were higher in the fluconazole (*n* = 4) and itraconazole (*n* = 5) groups compared to patients receiving posaconazole (*n* = 3). Posaconazole, fluconazole, and itraconazole are comparably effective in preventing invasive fungal infections in pediatric patients. Defining dose recommendations in these patients requires larger studies.

## Introduction

Invasive fungal infections belong to the most severe complications in patients with hemato-oncological malignancies during neutropenia after intensive chemotherapy, representing a leading cause for infectious mortality and morbidity in immunocompromised children. Immunocompromised pediatric patients with prolonged severe neutropenia resulting from high-dose chemotherapy have a high risk of acquiring invasive fungal infections, especially with *Aspergillus* spp. and *Candida* spp., so that a systemic antifungal prophylaxis is indicated [1–5]. Immunosuppression and high-dose steroids are additional risk factors for fungal infections [6]. Oral antifungal prophylaxis with other azoles, fluconazole, itraconazole, and voriconazole is preferentially used in pediatric patients after chemotherapy. However, only a few studies have been published on pediatric patients with hemato-oncological malignancies, while antifungal prophylaxis seems well described for adult patients [7–13]. Fluconazole shows particularly good efficacy against *Candida* infections but not with *Aspergillus* spp. [14–17]. Itraconazole has a broader range of efficacy in comparison to fluconazole, which includes *Aspergillus* spp. and other rare mold infections [18–20]. In pediatric patients with neutropenia after chemotherapy and hematopoietic stem cell transplantation (HSCT) who received fluconazole and itraconazole, fungal breakthrough infections occurred time and again [16, 21–25]. In the hospital where the present analysis was performed, the administration of fluconazole and itraconazole in pediatric patients with neutropenia after high-dose chemotherapy and HSCT was associated with isolated cases of breakthrough infections. Due to the favorable results of antifungal prophylaxis with the broad-spectrum triazole posaconazole in adults experiencing prolonged neutropenia or patients with graft-versus-host disease (GvHD) [11, 12, 26], the oral antifungal prophylaxis in pediatric patients after allogeneic HSCT was changed to posaconazole in 2007. During the present survey, the convincing results of these clinical studies in adults were also seen in the first 60 pediatric patients (<12 years of age), who received antifungal prophylaxis with posaconazole after allogeneic HSCT, regarding efficacy, safety, and feasibility [27]. This prompted another comparative analysis of three azoles as antifungal prophylaxis; itraconazole, voriconazole, and posaconazole. The retrospective analysis incorporated 150 immunocompromised pediatric patients after allogeneic HSCT and evaluated the comparable efficacy of these three azoles as antifungal prophylaxis [28].

Due to the excellent data from stem cell transplanted pediatric patients, the antifungal prophylaxis in pediatric patients after chemotherapy who experienced an anticipated prolonged neutropenia of at least 5 days was changed as well. Retrospectively, safety, feasibility, and initial data on the efficacy of posaconazole were compared with those of fluconazole and itraconazole in 93 pediatric patients and adolescents during the period of neutropenia after chemotherapy or during neutropenia caused by an underlying disease.

## Materials and methods

### Survey design

This analysis is a single-center, retrospective, non-randomized survey of 93 pediatric patients who received fluconazole, itraconazole, or posaconazole as oral antifungal monoprophylaxis during the phase of intensive induction, consolidation and re-induction chemotherapy, or during the period of neutropenia caused by an underlying disease. The survey was performed at the Department of Pediatric Hematology and Oncology, University Children’s Hospital Tuebingen, Germany. The patient cohorts consisted of pediatric patients and adolescents with hemato-oncological malignancies, starting chemotherapy between January 2006 and January 2013 (Table 1). The analysis included only pediatric patients who were diagnosed with prolonged neutropenia (neutrophil counts <500/μL; >5 days) when receiving antifungal prophylaxis. Antifungal monoprophylaxis with one of the three azoles began 24 h after the end of the chemotherapy cycle in pediatric patients with high-risk acute lymphoblastic leukemia (ALL), ALL relapse, acute myeloid leukemia (AML), AML relapse, non-Hodgkin lymphoma (NHL), Hodgkin lymphoma, osteosarcoma, Ewing sarcoma, and solid tumors, and ended at a minimum of one day before the start of chemotherapy. The treatment period was defined as the days when antifungal prophylaxis was orally administered with one of the three azoles. Pediatric patients with thalassemia major, aplastic anemia, or granulocytopenia were given antifungal monoprophylaxis during the whole period of neutropenia. Thirty-one of the 93 pediatric patients received antifungal prophylaxis with fluconazole at a dosage of 1 × 5 mg per kilogram of body weight per day (mg∙kg BW^−1^∙d^−1^) (maximum 1 × 400 mg/day), while 32 were given antifungal prophylaxis with itraconazole at a dosage of 2 × 5 mg∙kg BW^−1^∙d^−1^ (maximum 2 × 200 mg/day). Thirty other pediatric patients received antifungal prophylaxis with posaconazole 3 × 4 mg∙kg BW^−1^∙d^−1^ (maximum 3 × 200 mg/day). The primary objectives of this survey were to analyze the safety, feasibility, and efficacy of antifungal monoprophylaxis with fluconazole, itraconazole, or posaconazole for pediatric patients with hemato-oncological malignancies and neutropenia resulting from chemotherapy. The secondary objectives were to assess the incidence of invasive fungal infection with *Aspergillus* spp., *Candida* spp., or other fungal species. The observation period was defined as the time during the whole chemotherapy, including chemotherapy cycles. It started one day before the beginning of antifungal prophylaxis with one of the three azoles and ended three weeks after the last dosing or until the occurrence of a proven or probable fungal infection. For some pediatric patients, the observation period continued until shortly before the start of the conditioning regimen of the planned stem cell transplantation. The treatment period comprises days on which an antifungal monoprophylaxis with one of the three azoles, fluconazole, itraconazole, or posaconazole, was administered. The patients received fluconazole from 2006 to 2011, itraconazole from 2006 to 2012, and posaconazole from 2008 to 2013.

**Table 1:** Patient characteristics

Characteristic	Fluconazole (*n* = 31)	Itraconazole (*n* = 32)	Posaconazole (*n* = 30)	*p*-Value
	No. of patients (%)			
Gender				
Male	21 (67.7)	14 (43.8)	12 (40.0)	0.089
Female	10 (32.3)	18 (56.3)	18 (60.0)	(Chi-square)
Age group				
<6 years	8 (25.8)	7 (21.9)	9 (30.0)	
7–11 years	4 (12.9)	8 (25.0)	9 (30.0)	0.426
12 to <18 years	19 (61.3)	17 (53.1)	12 (40.0)	(Chi-square)
Diagnosis				
ALL	14 (45.2)	6 (18.8)	4 (13.3)	0.009
ALL relapse	1 (3.2)	7 (21.9)	9 (30.0)	0.018
AML	2 (6.5)	12 (37.5)	1 (3.3)	0.0003
AML relapse	0 (0.0)	3 (9.4)	3 (10.0)	0.215
MDS	0 (0.0)	0 (0.0)	1 (3.3)	0.346
NHL	6 (19.4)	1 (3.1)	3 (10.0)	0.114
Hodgkin lymphoma	3 (9.7)	1 (3.1)	0 (0.0)	0.163
Thalassemia major	0 (0.0)	0 (0.0)	1 (3.3)	0.346
Aplastic anemia	0 (0.0)	0 (0.0)	5 (16.7)	0.004
Granulocytopenia	0 (0.0)	0 (0.0)	1 (3.3)	0.346
Osteosarcoma	4 (12.9)	1 (3.1)	0 (0.0)	0.065
Ewing sarcoma	1 (3.2)	1 (3.1)	0 (0.0)	0.614
Solid tumor	0 (0.0)	0 (0.0)	2 (6.7)	0.117
Systemic corticosteroids in mg∙kg BW^−1^∙d^−1^				
Dexamethasone				0.708
≥2.0	0 (0.0)	0 (0.0)	0 (0.0	Fisher’s exact *t*
<2.0 but ≥1.0	0 (0.0)	0 (0.0)	1 (3.3)	
<1.0 but ≥0.5	9 (29.0)	10 (31.3)	8 (26.7)	
<0.5	8 (25.8)	5 (15.6)	5 (16.7)	
Prednisone/prednisolone				0.961
≥2.0	4 (12.9)	0 (0.0)	1 (3.3)	Fisher’s exact *t*
<2.0 but ≥1.0	4 (12.9)	1 (3.1)	1 (3.3)	
<1.0 but ≥0.5	0 (0.0)	0 (0.0)	2 (6.7)	
<0.5	0 (0.0)	0 (0.0)	1 (3.3)	
Methylprednisolone				*k* proportions test
≥2.0	0 (0.0)	0 (0.0)	2 (6.7)	0.117
<2.0 but ≥1.0	0 (0.0)	0 (0.0)	1 (3.3)	0.346
<1.0 but ≥0.5	0 (0.0)	0 (0.0)	0 (0.0)	1
<0.5	0 (0.0)	0 (0.0)	0 (0.0)	1

*ALL* acute lymphoblastic leukemia; *AML* acute myeloid leukemia; *MDS* myelodysplastic syndromes; *NHL* non-Hodgkin lymphoma

Statistical tests: “*k* proportions test” with XLSTAT 2013 or Chi-square test/Fisher’s exact test on contingency tables

### Assessment of safety and tolerance

For all of the 93 pediatric patients included in this survey, adverse events during the observation period and caused by one of the three azoles were graded according to the current United States National Cancer Institute’s Common Terminology Criteria for Adverse Events [29]. During the observation period, an analysis of liver and kidney parameters, as well as electrolytes, was performed. The analysis of hepatic toxicity included transaminases alanine aminotransferase (ALT, normal range ≤39 U/L) and aspartate aminotransferase (AST, normal range ≤39 U/L), cholestasis parameters total bilirubin (normal range ≤1.1 mg/dL) and direct bilirubin (normal range ≤0.3 mg/dL), and alkaline phosphatase (AP, normal range ≤320 U/L). The evaluation of kidney toxicity involved the examination of serum creatinine (normal range ≤0.7 mg/dL) and urea (normal range ≤46 mg/dL). In addition, an analysis of electrolytes potassium (normal range ≥3.4 mmol/L), calcium (normal range ≥2.0 mmol/L), and phosphate (normal range 1.1–1.5 mmol/L) was carried out. We assessed clinically relevant elevations of >1.5 and >2.5 times the normal values of hepatic and renal parameters, and a decrease of potassium values <3.4 mmol/L or <2.4 mmol/L, calcium values <2.0 mmol/L or <1.8 mmol/L, and phosphate values <1.1 mmol/L or <0.8 mmol/L. During the antifungal monoprophylaxis with one of the three azoles, fluconazole, itraconazole, or posaconazole, analysis of blood cell counts and hepatic and kidney function was performed a minimum of two times weekly. The evaluation of electrolytes was done up to a minimum of 3 weeks after the end of treatment with antifungal monoprophylaxis. Blood analyses were documented the day before the start of oral monoprophylaxis with one of the three azoles (baseline), as well as the maximum or minimum values during and at the end, defined as the last day of treatment with antifungal prophylaxis.

### Assessment of efficacy

All patients were monitored for clinical signs of infection, laboratory analyses, and radiological workup if indicated. The absence of clinically relevant symptoms such as fever or coughing and deviations of laboratory values beyond the normal range indicating a fungal infection were regarded as successful treatment. According to the European Organization for Research and Treatment of Cancer/National Institute of Allergy and Infectious Diseases Mycoses Study Group (EORTC/MSG) criteria of 2002 and 2008, invasive fungal infections were classified as proven, probable, and possible [30, 31]. The galactomannan antigen was measured until the occurrence of fever with duration of 72 h ≥38.5 °C by the Platelia™ *Aspergillus* enzyme-linked immunosorbent assay according to the manufacturer’s protocol (Bio-Rad Laboratories, Munich, Germany). The non-occurrence of clinical or microbiological signs of invasive fungal breakthrough infection during the observation period with antifungal monoprophylaxis was considered as successful antifungal treatment.

### Statistical analysis

This retrospective analysis included 93 pediatric patients who received antifungal monoprophylaxis with fluconazole, itraconazole, or posaconazole after chemotherapy or during immunosuppressant therapy. The statistical comparison of differences between the results and normal range values of liver and kidney parameters and electrolytes was performed by one-sample *t*-tests. This took into account the 95 % confidence intervals. The inferential statistical analysis between the baseline values and maximum and minimum parameters, as well as the parameters at the cessation of antifungal monoprophylaxis, was performed using the Friedman two-way analysis of variance (ANOVA) by ranks. Values were only considered significant if they were above the age-adjusted reference. The analysis of hepatic and renal function parameters are presented as mean values + standard deviation (SD). Differences in the frequencies of medication groups, for example at primary diagnosis, application of steroids, or incidence of breakthrough infections when comparing the three patient groups (with *n* = 31, *n* = 32, and *n* = 30 patients), were tested by the *k* proportions test. Medications with available contingency tables for up to four classes were first calculated using Fisher’s exact test. This was followed by a *k* proportions test for each individual class (i.e., first a global test for the entire frequency table and then separate tests for each class). For statistical comparison of the three groups regarding clinical and laboratory adverse events during treatment with antifungal prophylaxis, the arithmetic mean values of the three medication groups with a one-way ANOVA were tested for differences. Values of *p* < 0.05 (*), *p* < 0.01 (**) and *p* < 0.001 (***) were defined as significant. XLSTAT 2013 (AddinSoft, Paris, France) or GraphPad Prism® Version 5.04 for Windows (GraphPad Software, La Jolla, CA, USA) were used for the statistical analyses. Graphs were created with GraphPad Prism® Version 5.04 for Windows.

## Results

### Patient characteristics

This single-center analysis involved the evaluation of 93 pediatric patients with hemato-oncological malignancies aged between 9 months and 17.7 years who received an antifungal monoprophylaxis with fluconazole, itraconazole, or posaconazole during a period of prolonged neutropenia (>5 days). The median age in the fluconazole group was 14 years (range 1–17.5 years), while the itraconazole and posaconazole groups had medians of 12 years (range 1–17.7 years) and 11 years (range 9 months to 17.4 years), respectively. Significant differences in clinical characteristics were noted regarding the number of children with ALL (*p* = 0.009), ALL relapse (*p* = 0.018), and AML (*p* = 0.0003) included in this analysis, but no significant differences were found in the AML relapse group (*p* = 0.215). The percentage of all these types of diagnoses of the fluconazole group (i.e., 54.8 % acute leukemia; 17 of 31 patients) was comparable (*p* = 0.1611) to the posaconazole group (56.7 %; 17 of 30 patients), but significantly different (*p* = 0.0054) from the itraconazole group (87.5 %; 28 of 32 patients; see Table 1). Information on chemotherapy regimens is presented in Table 2.

**Table 2:** Treatment regimens

Characteristics		Fluconazole (*n* = 31)	Itraconazole (*n* = 32)	Posaconazole (*n* = 30)	*p*-Value
		No. of patients (%)			
Alkylating antineoplastic agent	CPM	24 (77.4)	8 (25.0)	8 (26.7)	<0.0001
	Dacarbazine	2 (6.5)	0 (0.0)	1 (3.3)	0.350
	Ifosfamide	10 (32.3)	9 (28.1)	6 (20.0)	0.548
	Melphalan	0 (0.0)	0 (0.0)	1 (3.3)	0.346
Anthracyclines	Daunorubicin	12 (38.7)	14 (43.8)	8 (26.7)	0.360
	Doxorubicin	13 (41.9)	3 (9.4)	1 (3.3)	0.0001
	Idarubicin	2 (6.5)	15 (46.9)	1 (3.3)	<0.0001
Antimetabolites					
Folic acid analogues	Methotrexate	23 (74.2)	10 (31.3)	6 (20.0)	<0.0001
Purine analogues	Clofarabine	0 (0.0)	1 (3.1)	3 (10.0)	0.145
	Fludarabine	1 (3.2)	4 (12.5)	2 (6.7)	0.369
	Nelarabine	0 (0.0)	0 (0.0)	1 (3.3)	0.346
	Tioguanine	3 (9.7)	7 (21.9)	3 (10.0)	0.282
	6-Mercaptopurine	9 (29.0)	5 (15.6)	5 (16.7)	0.345
Pyrimidine analogues	Cytarabine	22 (71.0)	26 (81.3)	15 (50.0)	0.028
	2-CDA	1 (3.2)	3 (9.4)	0 (0.0)	0.179
Enzymes	Asparaginase	12 (38.7)	10 (31.3)	9 (30.0)	0.735
Platinum complex compounds	Carboplatin	1 (3.2)	0 (0.0)	0 (0.0)	0.364
	Cisplatin	4 (12.9)	1 (3.1)	0 (0.0)	0.065
Topoisomerase inhibitors	Etoposide	13 (41.9)	17 (53.1)	6 (20.0)	0.025
	Mitoxantrone	2 (6.5)	10 (31.3)	0 (0.0)	0.001
	Topotecan	1 (3.2)	0 (0.0)	0 (0.0)	0.364
Vinca alkaloids	Vinblastine	0 (0.0)	0 (0.0)	1 (3.3)	0.346
	Vincristine	19 (61.3)	11 (34.4)	10 (33.3)	0.042
	Vindesine	8 (25.8)	6 (18.8)	5 (16.7)	0.648
Intrathecal medication	Cytarabine	14 (45.2)	24 (75.0)	13 (43.3)	0.018
	methotrexate	22 (71.0)	26 (81.3)	12 (40.0)	0.002
	Predniso(n)/lon	14 (45.2)	24 (75.0)	11 (36.7)	0.006
Antibodies	ATG	0 (0.0)	0 (0.0)	1 (3.3)	0.346
	Blinatumomab	0 (0.0)	0 (0.0)	1 (3.3)	0.346
	CD19 antibody	0 (0.0)	1 (3.1)	0 (0.0)	0.382
	Rituximab	0 (0.0)	0 (0.0)	2 (6.7)	0.117
Tyrosine kinase inhibitors	Nilotinib	0 (0.0)	0 (0.0)	1 (3.3)	0.346
	Imatinib	0 (0.0)	1 (3.1)	0 (0.0)	0.382

*ATG* anti-thymocyte globulin; *CPM* cyclophosphamide; *CSA* ciclosporin A; *2-CDA* 2-chloro-2′-deoxyadenosine

Statistical tests: *k* proportions test with XLSTAT 2013

### Treatment and observation period

The median observation periods were 135 days (range 47–212 days) for the fluconazole group, 104 days (range 47–186) for the itraconazole group, and 107 days (range 28–236 days) for the posaconazole group. The median treatment period was 116 days (range 25–189 days) for the fluconazole group, 85 days (range 23–164 days) for the itraconazole group, and 86 days (range 6–214 days) for the posaconazole group.

### Mortality

Seven (7.5 %) of the 93 pediatric patients died during the observation period. None of the patients included in the analysis died of invasive fungal infection. Four patients died due to a relapse of their primary diagnosis. Two patients died of multiple organ failure due to sepsis, while another one died of cardiac failure after chemotherapy.

### Neutropenia

The count of prolonged neutropenic periods (neutrophil counts <500/μL; >5 days) were similar in all three groups, with medians of two periods (range 1–7) in the fluconazole group, two periods (range 1–6) in the itraconazole group, and two periods (range 1–6) in the posaconazole group. The median durations of the prolonged neutropenic periods were 7 days (range 5–47 days), 9 days (range 6–78 days), and 7 days (range 5–214 days), with means of 8.9 ± 3.2, 12.6 ± 6.1, and 17.4 ± 11.4, respectively.

### Adverse events

The spectrum of drug-related adverse events caused by one of the three azoles was characterized primarily by gastrointestinal symptoms such as abdominal pain, nausea, or diarrhea in all three groups. Other adverse events in the fluconazole group were headache, while patients in the itraconazole group experienced fever and exanthema (Table 3). Adverse events that may be related to antifungal treatment were observed in four patients in the fluconazole group, in five patients in the itraconazole group, and in three patients in the posaconazole group. The differences between these three groups were not significant (*p* = 0.804). Due to adverse events, oral antifungal prophylaxis was withdrawn for two of the four patients treated with fluconazole, for three of the five patients treated with itraconazole, and for one of the three patients treated with posaconazole.

**Table 3:** Clinical and laboratory adverse events during antifungal prophylaxis

Characteristics	Fluconazole (*n* = 31)	Itraconazole (*n* = 32)	Posaconazole (*n* = 30)	*p*-Value
	No. of patients (%)			
Drug-related adverse events				
Clinical (total)	4 (12.9)	5 (15.6)	3 (10.0)	0.804
Fever	0 (0.0)	1 (3.1)	0 (0.0)	0.382
Headache	1 (3.2)	0 (0.0)	0 (0.0)	0.364
Nausea	1 (3.2)	1 (3.1)	1 (3.3)	0.999
Diarrhea	2 (6.5)	1 (3.1)	0 (0.0)	0.362
Exanthema	0 (0.0)	1 (3.1)	0 (0.0)	0.382
Abdominal pain	0 (0.0)	1 (3.1)	2 (6.7)	0.338
Increase in alanine aminotransferase				
>1.5 × normal value 39 U/L	8 (25.8)	7 (21.8)	3 (10.0)	0.267
>2.5 × normal value 39 U/L	4 (12.9)	4 (12.5)	5 (16.7)	0.874
Increase in aspartate aminotransferase				
>1.5 × normal value 39 U/L	4 (12.9)	2 (6.6)	5 (16.7)	0.435
>2.5 × normal value 39 U/L	3 (9.7)	3 (9.4)	1 (3.3)	0.571
Increase in alkaline phosphatase				
>1.5 × normal value 320 U/L	1 (3.2)	0 (0.0)	0 (0.0)	0.364
>2.5 × normal value 320 U/L	0 (0.0)	0 (0.0)	0 (0.0)	1.0
Increase in total bilirubin				
>1.5 × normal value 1.1 mg/dl	1 (3.2)	2 (6.3)	2 (6.7)	0.807
>2.5 × normal value 1.1 mg/dl	0 (0.0)	0 (0.0)	0 (0.0)	1.0
Increase in direct bilirubin				
>1.5 × normal value 0.3 mg/dl	2 (6.5)	3 (9.4)	2 (6.7)	0.887
>2.5 × normal value 0.3 mg/dl	0 (0.0)	3 (9.4)	1 (3.3)	0.177
Increase in creatinine				
>1.5 × normal value 0.7 mg/dl	1 (3.2)	0 (0.0)	1 (3.3)	0.585
>2.5 × normal value 0.7 mg/dl	0 (0.0)	0 (0.0)	0 (0.0)	1.0
Increase in urea				
>1.5 × normal value 46 mg/dl	1 (3.2)	0 (0.0)	0 (0.0)	0.364
>2.5 × normal value 46 mg/dl	0 (0.0)	0 (0.0)	0 (0.0)	1.0
Decrease in potassium				
<3.4 mmol/L	2 (6.5)	3 (9.4)	2 (6.7)	0.867
<2.4 mmol/L	0 (0.0)	0 (0.0)	0 (0.0)	1.0

Statistical tests: *k* proportions test with XLSTAT 2013

### Efficacy analysis

All 93 pediatric patients were included in the efficacy analysis (Table 4). Overall, there were two invasive fungal breakthrough infections in the fluconazole group, three in the itraconazole group, and one in the posaconazole group, according to the EORTC guidelines [30, 31]. In each of the three azole groups, there was one proven fungal infection. Probable infections did not occur. Possible fungal infections were seen in one patient in the fluconazole group and two patients in the itraconazole group.

**Table 4:** Breakthrough invasive fungal infections during antifungal prophylaxis

Characteristics	Fluconazole (*n* = 31)	Itraconazole (*n* = 32)	Posaconazole (*n* = 30)	*p*-Value
	No. of patients			
Invasive fungal infection: total no.	2	3	1	0.626
*Aspergillus* spp.				
Proven	1	1	1	0.999
Probable	–	–	–	
Possible	1	2	–	0.380
*Candida* spp.				
Proven	–	–	–	
Probable	–		–	
Possible	–	–	–	
Others	–	–	–	

Statistical tests: *k* proportions test with XLSTAT 2013

In the fluconazole group, one patient with high-risk ALL, who presented with neutropenia and coughs, experienced an *Aspergillus* spp. infection with typical fungus infiltrates in the lungs and brain. The galactomannan antigen was positive in more than two consecutive blood analyses. There was also positive microbiological detection in the sputum. An antifungal combination therapy with caspofungin, voriconazole, and liposomal amphotericin B was given. Both the pulmonary nodule and two cerebral foci were resected. After clinical discharge, antifungal therapy was given with posaconazole for a further six months. During this time, there were no more signs of invasive fungal infection. A possible invasive infection was observed in this group for one patient suffering from ALL. The computed tomography (CT) scan showed foci that were suspected to be fungi herds. However, galactomannan antigen was negative.

In the itraconazole group, one patient with AML experienced thoracic pain and fever during the neutropenia period between chemotherapy cycles. The CT scan showed an *Aspergillus* pneumonia with a halo sign and increase in galactomannan antigen. The patient was treated with a triple antifungal prophylaxis including caspofungin, voriconazole, and liposomal amphotericin B. After resection of the pulmonary foci, antifungal therapy was continued with antifungal monotherapy with liposomal amphotericin B. The patient received oral antifungal therapy with posaconazole after clinical discharge for a further five months. During this time, there was no sign of invasive fungal infection. In this group, two possible fungal infections occurred in patients with AML who underwent chemotherapy cycles. Typical fungus lung infiltrates were found on the CT scan. In both patients, the galactomannan antigen was not detectable. A lung lavage was not performed.

In the posaconazole group, one patient with granulocytopenia experienced fever and pain in the right lower abdomen. Up to that point, the pediatric patient had been treated with posaconazole antifungal monoprophylaxis for a period of 104 days. An appendectomy was performed and its biopsy material showed evidence of an *Aspergillus* infection. The galactomannan antigen and blood cultures were negative during the entire period when antifungal prophylaxis with posaconazole was given. The antifungal prophylaxis was continued with posaconazole in therapeutic dosage for another 110 days after the appendectomy until the planned stem cell transplantation. At the time of HSCT, intravenous antifungal prophylaxis was given with liposomal amphotericin B during the conditioning regimen and on day 1 after HSCT with caspofungin. Shortly before clinical discharge, there was a change to antifungal oral monoprophylaxis with posaconazole until reaching immune reconstitution (CD3+ cells 200/μL and CD4+ cells to 100/μL; until day 120 after transplantation). There were no more signs of an invasive fungal infection during the entire period after stem cell transplantation. A possible invasive fungal infection did not occur in this patient group.

A probable invasive infection did not occur in any of the three azole groups.

### Safety and tolerance

**Fig. 1:**
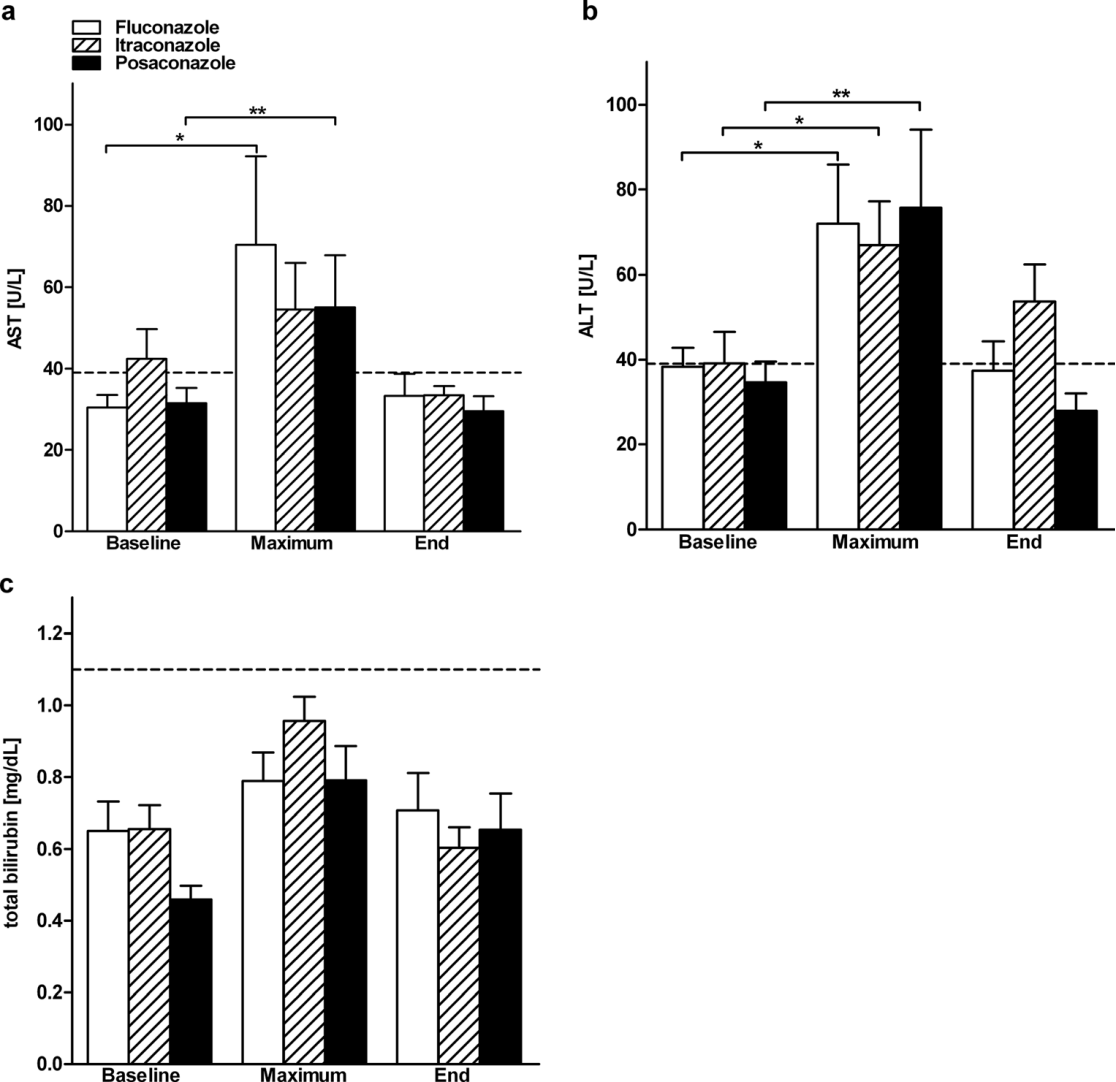
Hepatotoxicity. The data show mean values + standard deviation (SD) of transaminases and total bilirubin on the day before the start of oral antifungal prophylaxis (*Baseline*) and maximum values during (*Maximum*) and at the end (*End*) of treatment with fluconazole, itraconazole, and posaconazole. Normal values are indicated by the *dotted lines*. **a** Mean + SD of serum concentration of aspartate aminotransferase (*AST*) (normal <39 U/L). ); *p*-values maximum compared to baseline: fluconazole (*p* = 0.049), itraconazole (*p* > 0.05), and posaconazole (*p* = 0.005). **b** Mean + SD of serum concentration of alanine aminotransferase (*ALT*) (normal <39 U/L); *p*-values maximum compared to baseline: fluconazole (*p* = 0.031), itraconazole (*p* = 0.015), and posaconazole (*p* = 0.007). **c** Mean + SD of serum concentration of total bilirubin (normal <1.1 mg/dL); *p*-values maximum compared to baseline: fluconazole (*p* > 0.05), itraconazole (*p* > 0.05), and posaconazole (*p* > 0.05). None of the changes in the ALT, AST, or total bilirubin serum concentrations were clinically relevant. Statistical significance was tested by the Wilcoxon matched-pairs signed-rank test. *p < 0.05; **p < 0.01

**Fig. 2:**
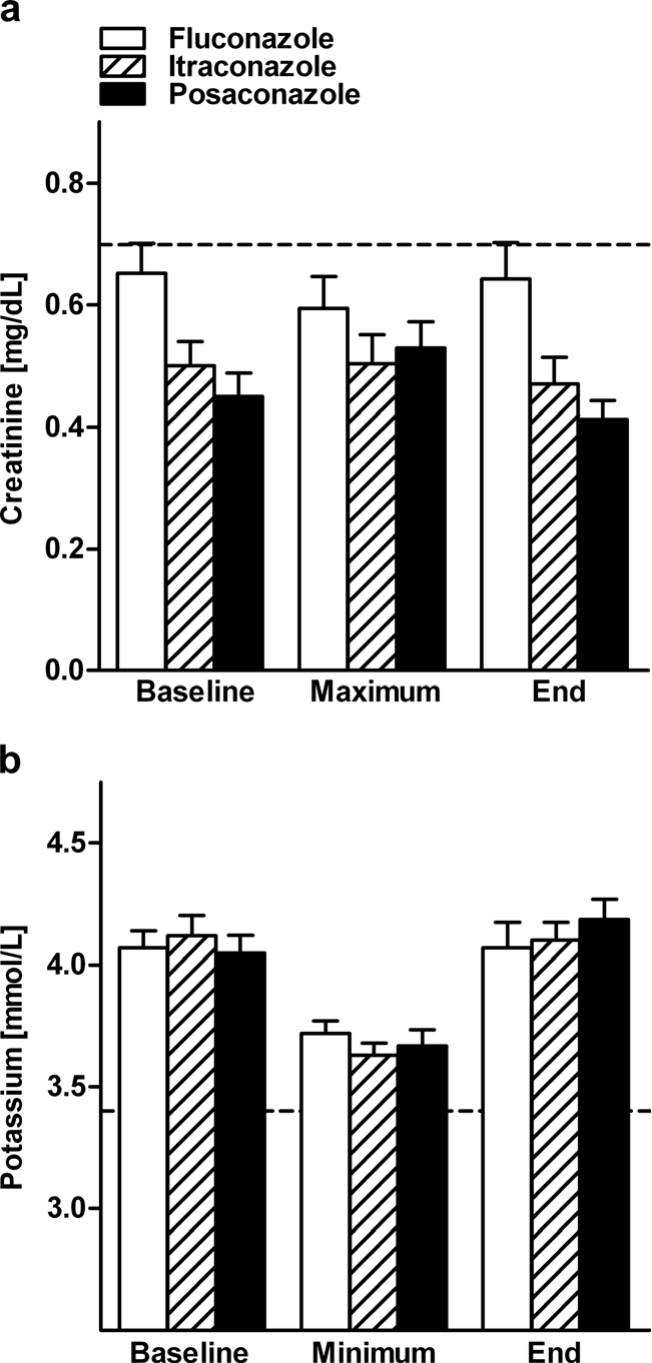
Nephrotoxicity. The data show mean values + standard deviation (SD) of renal parameters on the day before the start of oral antifungal prophylaxis (*Baseline*) and maximum (*Maximum*) or minimum (*Minimum*) values during and at the end of fluconazole, itraconazole, and posaconazole treatment. Normal values are indicated by the *dotted lines*. **a** Mean + SD of serum concentration of creatinine (normal <0.7 mg/dL). **b** Mean + SD of serum concentration of potassium (normal >3.4 mmol/L). Statistical significance was tested by the Wilcoxon matched-pairs signed-rank test

The blood analysis of liver and kidney parameters showed a significant increase beyond the upper normal limit of ALT between baseline and maximum in all three groups, i.e., fluconazole (*p* = 0.031), itraconazole (*p* = 0.015), and posaconazole (*p* = 0.007), and a significant increase of AST between baseline and maximum in the fluconazole (*p* = 0.049) and posaconazole groups (*p* = 0.005) (Fig. 1). An increase in ALT of >1.5 and 2.5 times the normal value occurred in a total of 12 pediatric patients in the fluconazole group, 11 pediatric patients in the itraconazole group, and eight pediatric patients in the posaconazole group. AST >1.5 and 2.5 times the normal value occurred in a total of seven pediatric patients in the fluconazole group, five pediatric patients in the itraconazole group, and six pediatric patients in the posaconazole group (Table 3). The total and direct bilirubin and AP showed no significant increase above the normal values during antifungal prophylaxis with any of the three azoles. Kidney parameters creatinine and urea were not significantly elevated beyond the upper limit of the normal range during antifungal monoprophylaxis in any of the treatment groups (Fig. 2). In all three groups, there was no significant decrease in potassium during antifungal prophylaxis with any of the three azoles.

## Discussion

The goal of antifungal prophylaxis treatment in pediatric patients with prolonged neutropenia is to prevent invasive fungal infections, thereby minimizing mortality. While several evidence-based guidelines are available for adults with hemato-oncological malignancies [32–36], there are only limited data on pediatric patients [37, 38]. Due to the favorable results of oral antifungal prophylaxis with posaconazole in adults with myelodysplastic syndromes (MDS) and AML in randomized clinical trials involving more than 300 high-risk patients with neutropenia and more than 300 transplanted patients with GvHD [11, 12], we have also been using oral antifungal prophylaxis with posaconazole at our clinic since 2007. The excellent results in the first 60 pediatric patients treated with posaconazole after HSCT and comparably successful analysis of posaconazole with itraconazole and voriconazole in 150 pediatric patients after allogeneic HSCT [27, 28] were the decisive factors to perform this retrospective analysis. This analysis, which includes 93 pediatric patients with a median age of 12 years, shows that the incidence of invasive fungal infections in pediatric patients was low under each of the three azoles posaconazole, itraconazole, and fluconazole.

In each of the three treatment groups, there was one proven fungal infection, but no occurrence of probable fungal infections. Possible fungal infections were observed in both the itraconazole and fluconazole groups, but not in the posaconazole group. The occurrence of proven fungal infection in all three groups raises the question as to whether the dosage and resorption of the extended-spectra azoles were adequate, as regular assessment of trough levels had not been performed.

The data on pediatric patients regarding the efficacy, safety, and tolerability of posaconazole are mainly confined to analyses of small numbers of patients. A multicenter retrospective analysis, which examined the treatment of proven or probable invasive fungal infections, showed a remission in 9 of the 15 pediatric patients under salvage therapy with posaconazole [39]. Another retrospective study in 15 children with proven (*n* = 1), probable (*n* = 10), or possible (*n* = 1) fungal infection after HSCT or neutropenia after chemotherapy showed that posaconazole, as secondary prophylaxis, is to be regarded as being safe and well tolerated by children [40]. After 90 days of treatment with posaconazole, significantly improved radiological results were shown in nine pediatric patients. Twelve months after the onset of fungal infection in 12 patients, the survival rate was 91.67 %.

In a single-center retrospective study, 2 of 53 pediatric patients given intravenous and then oral antifungal prophylaxis with itraconazole after HSCT experienced a proven fungal infection [21]. A comparison of the efficacy of itraconazole, voriconazole, fluconazole, and posaconazole as antifungal prophylaxis in adults showed that invasive fungal infections occurred the least often (in 3 % of cases) in patients given posaconazole, while patients treated with fluconazole experienced invasive fungal infections at the highest rate (25 % of cases) [41]. More effective prevention of invasive fungal infections and, thus, an improvement in the survival rate was observed with posaconazole antifungal prophylaxis in 2 % of 304 adult patients treated, compared to itraconazole and fluconazole, where fungal infection occurred in 8 % of the cases [11].

The results of the present survey show that clinical, potentially azole-related adverse events are higher in the fluconazole (*n* = 4) and itraconazole (*n* = 5) groups than in the posaconazole group (*n* = 3). However, these results are not statistically significant.

Mainly gastrointestinal adverse events such as nausea, diarrhea, and abdominal pain occurred in all three groups with the same frequency. Fortunately, no azole-associated peripheral neuropathy side effect was observed in any of the patients from the three groups. Similar results have been reported in several studies on long-term triazole therapy [42, 43]. These results can probably be attributed to the cessation of antifungal prophylaxis with one of the azoles 24 h before the start of chemotherapy and 24 h after chemotherapy with the vinca alkaloid.

In a multicenter randomized trial with adults, the comparison of itraconazole with fluconazole showed that itraconazole is more effective as a long-term prophylaxis. However, while taking itraconazole (both intravenous and oral), gastrointestinal side effects occurred significantly (*p* = 0.02) more often than with fluconazole recipients [16].

In a randomized, double-blind trial of 600 patients after stem cell transplantation with GvHD, antifungal prophylaxis of invasive fungal infections with oral posaconazole in 301 patients was compared to oral fluconazole in 299 patients. Gastrointestinal disorders such as diarrhea, nausea, and abdominal pain occurred more often in the fluconazole group (18 %) than in the posaconazole group (14 %) [44]. In an analysis using data derived from 12 clinical studies of fluconazole as prophylaxis or treatment for a variety of fungal infections including 562 predominantly immunocompromised pediatric patients, the most common side effects were associated with the gastrointestinal tract (7.7 %) [44]. In studies which analyzed the safety and tolerability of posaconazole in pediatric patients, side effects such as nausea and vomiting occurred at varying frequencies [27, 39, 40].

In the present analysis, the transaminases ALT and AST increased significantly beyond the normal ranges during antifungal prophylaxis, whereas total bilirubin, direct bilirubin, creatinine, urea, and potassium showed no significant changes during the treatment. Changes in the transaminases of the itraconazole and posaconazole groups in the present investigation correspond to prior observations made at our hospital during antifungal prophylaxis with these azoles [27, 28]. In a retrospective single-center study, 10 (18.9 %) out of the 53 pediatric patients that received antifungal prophylaxis with itraconazole after HSCT were found to have at least a doubling of the baseline value of AST [21]. In different published studies with immunocompromised pediatric and juvenile patients suffering from hemato-oncological malignancies, transaminases ALT and AST were also increased under antifungal prophylaxis and therapy with fluconazole in 6–18 % of the recipients [44].

In summary, in the present survey, the incidence of invasive fungal infections in pediatric patients was similarly low under each of the azoles during the observation period. One case of proven aspergillosis occurred in each group. While this can be explained in the case of fluconazole, it remains an open question as to why extended-spectra azoles were not capable of preventing these breakthrough infections. Inadequate dosing and/or inadequate trough levels due to malabsorption may have contributed to the treatment failures. A few cases of possible invasive fungal infection under fluconazole and itraconazole, but none under posaconazole, were observed. Potentially clinical drug-related adverse events were similar in the three treatment groups. Laboratory parameters were comparable during fluconazole, itraconazole, and posaconazole treatment. Larger cohorts and a prospective setting are needed in order to establish the efficacy of prophylactic use of extended-spectra azoles in this patient cohort.

## Abbreviations

HSCT: Hematopoietic stem cell transplantation
ALL: Acute lymphoblastic leukemia
ALT: Alanine aminotransferase
AML: Acute myeloid leukemia
ANOVA: Analysis of variance
AP: Alkaline phosphatase
AST: Aspartate aminotransferase
CT: Computed tomography
EORTC/MSG: European Organization for Research and Treatment of Cancer/Allergy and Infectious Diseases Mycoses Study Group
GvHD: Graft-versus-host disease
h: Hour(s)
MDS: Myelodysplastic syndromes
mg/dL: Milligrams per deciliter
mg∙kg BW^−1^∙d^−1^: Milligrams per kilogram of body weight per day
mmol/L: Millimol per liter
NHL: Non-Hodgkin lymphoma
U/L: Units per liter
